# Squamous cell carcinoma developed in a chronic radiation-induced chest wall ulcer that is difficult to undergo thorough preoperative histological examination

**DOI:** 10.1016/j.ijscr.2020.05.081

**Published:** 2020-06-17

**Authors:** Masamitsu Kuwahara, Satoshi Yurugi, Junji Ando, Mika Takeuchi, Riyo Miyata, Masayuki Harada, Yasumitsu Masuda, Saori Kanagawa

**Affiliations:** Division of Plastic Surgery, Nara Medical University Hospital, 840 Shijocho, Kashihara, Nara 634-8522, Japan

**Keywords:** Radiation ulcer, Radiation injuries, Radiation induced skin cancer

## Abstract

**Introduction:**

We report a case in which squamous cell carcinoma (SCC) developed in a large chronic radiation-induced thoracic ulcer after flap surgery in areas where preoperative histological examinations are difficult.

**Presentation of case:**

The patient was a 75-year-old female. She had undergone resection and radiotherapy for left breast cancer 15 years earlier. Six years ago, the ulcer expanded from the subclavian to xiphoid levels, exposing the lung and pericardium. A histopathological examination, which avoided the lung and pericardium, was performed. Inflammation was diagnosed. We reconstructed the chest wall with a pedicled rectus abdominis flap. Eighteen months later, three verrucous tissue-lined fistulas formed. A histological examination revealed well-differentiated SCC. Six months later, the patient died of massive bleeding from a fistula.

**Discussion:**

It is unclear exactly when the SCC occurred. As three fistulas formed at the margins of the flap around the pericardium, we suspect that the cancer developed within or near the pericardial region.

We need to reflect on the lack of a thorough biopsy. As no pericardial biopsy was performed, we should have asked a thoracic/cardiac surgeon to conduct a biopsy during the debridement operation. If the tumor had been localized to the pericardium, it could have been removed.

**Conclusion:**

It is necessary to consider the best method for performing the most thorough histological examination possible, even in areas where histological examinations are difficult, as all ulcers can contain tumors.

## Highlights

• Squamous cell carcinoma (SCC) arose in a large chronic radiation-induced thoracic ulcer which exposing the lung and pericardium after flap surgery.
• Preoperative histopathological examination point indicated Inflammation. But after flap surgery, squamous cell carcinoma was diagnosed from a fistula at the margin of the flap.
• We should have asked a thoracic/cardiac surgeon to conduct a biopsy at the pericardium during the debridement operation before flap covering.
• It is necessary to consider the best method for performing the most thorough histological examination possible, even in areas where histological examinations are difficult, as all ulcers can contain tumors.

## 1 Introduction

Radiotherapy reduces the risk of the local recurrence of primary cancer and increases survival rates, but it can have severe adverse effects on normal tissue, e.g., it can cause radiation-induced ulcers.

Radiation-induced ulcers are classified into acute or chronic ulcers. Chronic type radiation-induced ulcers can appear up to 10 years after irradiation and are initiated by damage to the endothelial microvessels [1,2]. This damage eventually causes non-healing ulcers and soft tissue necrosis.

On the other hand, it is known that radiation and chronic wounds can induce skin cancer [[3], [4], [5]]. SCC that arises in a chronic wound (Marjolin's ulcer) is the most common type of malignancy [4]. But BCC is the most common type of malignancy affecting radiation wounds and SCC is rare [5]. In breast cancer, secondary cutaneous squamous cell carcinoma after post-surgical radiation is very rare [6,7].

As for the surgery of such chronic wound, biopsy is generally performed before treatment to deny the presence of tumor [8,9].

We also intended to treat chest wall radiation ulcer according to this treatment policy but failed to detect SCC that might have already developed.

We report the case of a patient in whom SCC was found in a chronic radiation-induced chest wall ulcer after flap surgery in areas where preoperative thorough histological examinations are difficult.

This work has been reported in line with the SCARE criteria [10].

## 2 Patient

The patient was a 75-year-old female. She had developed large radiation ulcer on her left chest. she visited another hospital and underwent histopathological examination on the center of the ulcer, which avoided the lung and pericardium. The examination only revealed inflammation.

The course before she received a histopathological examination was as follows: She had undergone resection and radiotherapy (dose unknown) for left-sided breast cancer 15 years earlier. She said that the radiated wound took a year to heal. six years ago, ulcer formed and expanded, but she treated it conservatively by herself.

**Fig. 1:**
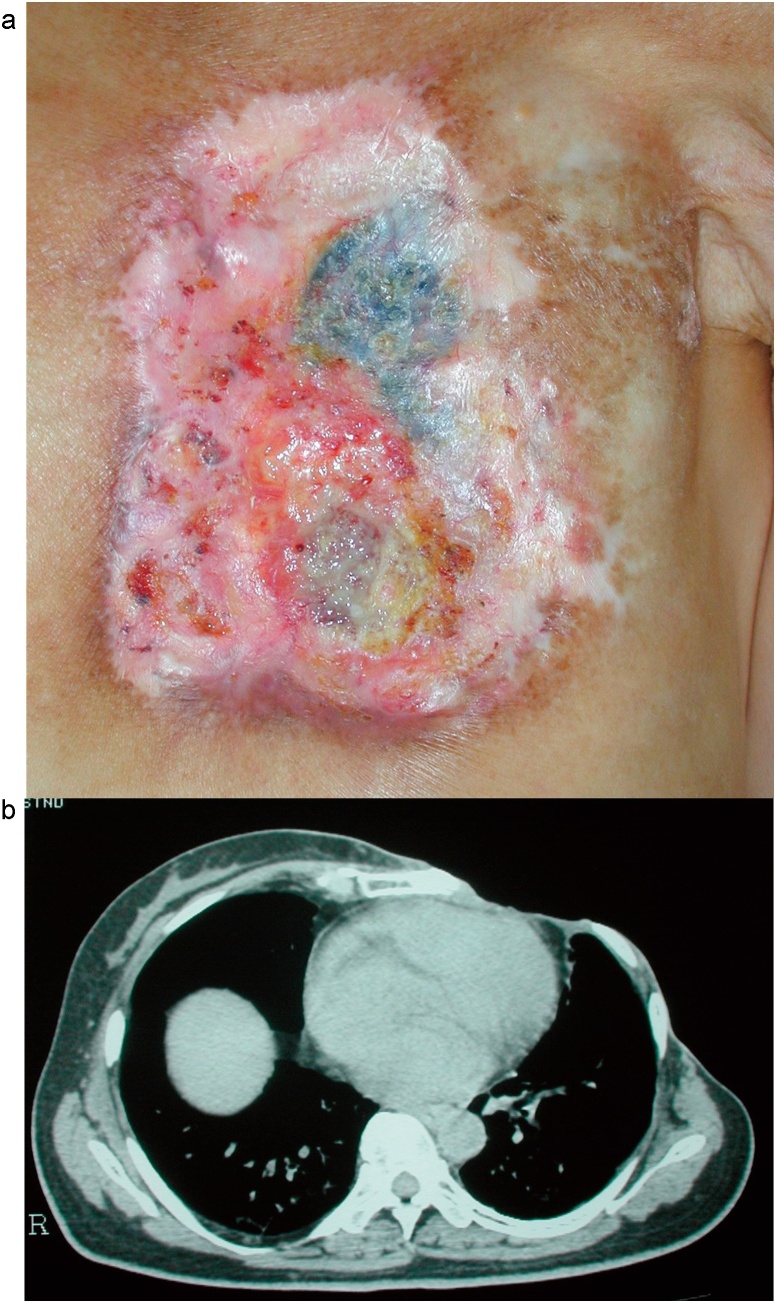
(a) Findings obtained when the patient visited our hospital. The ulcer affecting the left chest wall extended from the subclavian to xiphoid levels. The third, fourth, fifth, and sixth costal cartilages and bones were missing, and parts of the lung and pericardium could be seen. (b) Computed tomography image. The ulcer was deep enough to expose the pericardium. Computer tomography image. There is only a small gap between the ulcer and the heart.

After the histopathological examination, the patient was referred to our hospital. The ulcer in the left chest wall extended from the subclavian to xiphoid levels. The third, fourth, fifth and sixth costal cartilages and bones were missing, and parts of lung and pericardium could be seen　(Fig. 1a, b).

We debrided the ulcer with the help of a thoracic surgeon and reconstructed the chest wall with Marlex mesh and a pedicled rectus abdominus flap (Fig. 1). The wound-healing process and postoperative course was uneventful.

One year later after the first operation and 11 months before the last follow up, a fistula formed. We suspected that it had been caused by a mesh-related infection and removed the mesh.

Bacterial cultures obtained preoperatively and after second mesh-removal procedure only detected methicillin-sensitive *Staphylococcus aureus* and normal *Pseudomonas aeruginosa*.

**Fig. 2:**
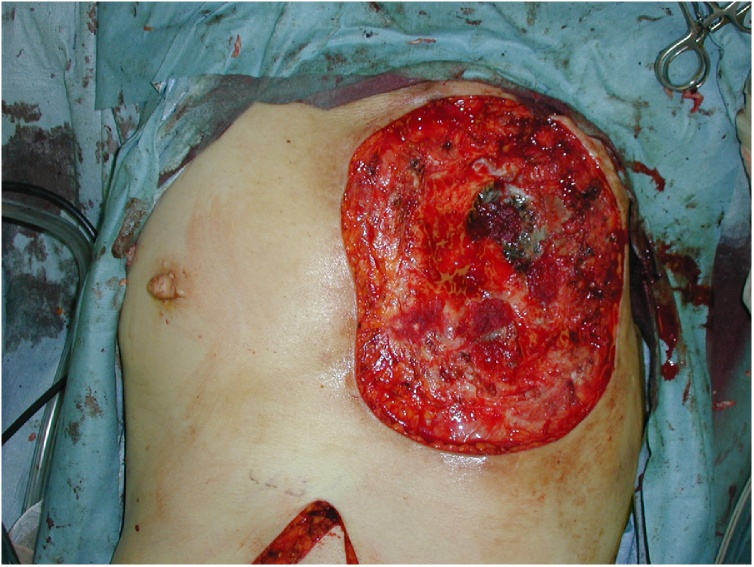
Intraoperative image obtained just after debridement.

**Fig. 3:**
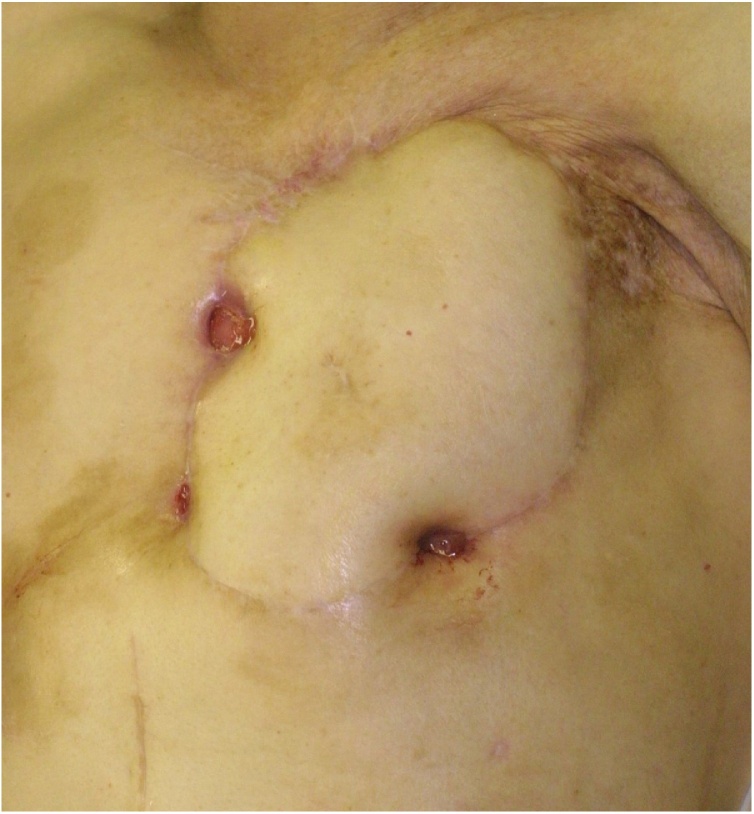
Findings obtained one year and four months later. After the first surgery, three fistulas, which were lined by verrucous tissue, formed.

Four months later mesh removal and 7 months before the last follow up, three fistulas which were lined by verrucous tissue, formed (Fig. 2). A histological examination of the verrucous tissue revealed well-differentiated SCC (Fig. 3). We consulted heart surgeon about the possibility for a further operation, but the patient refused to consider further surgery or treatment because she had no relatives to depend on. Six months later, she was admitted with general fatigue and died due to massive bleeding from a fistula.

## 3 Discussion

AS the hospital that treated and histologically examined our patient has closed, the histology of her breast cancer and the dose of radiation administered could not be determined. Among all breast cancers, SCC is rare (it accounts for <0.1% of breast cancer cases) and has a 5-year survival rate of 63.5% [11].

**Fig. 4:**
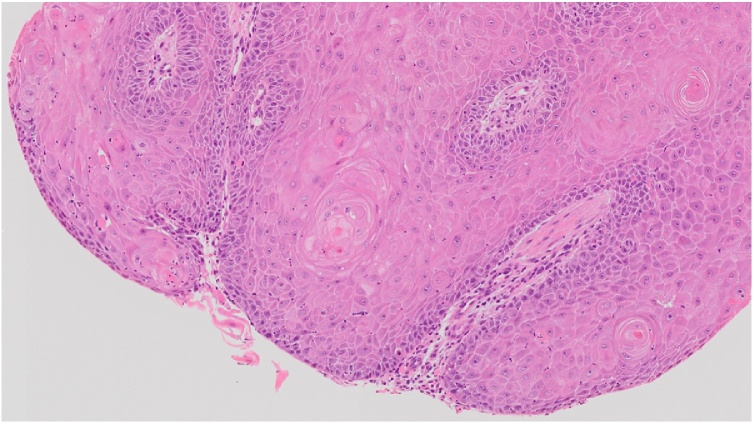
Histological examination of the verrucous tissue Well-differentiated squamous cell carcinoma was detected. Prominent keratinocyte proliferation and pronounced keratinization were observed. In addition, the tumor cells had atypical, irregularly sized nuclei and exhibited mitosis. Some cancer pearl formation was seen.

In addition, most of the primary SCCs in the breast are moderately or poorly differentiated type and tend to be aggressive [12]. Specimens of our patients are considered to be well differentiated type (Fig. 4). There was no report that indicate late recurrence of primary SCC in breast. Therefore, we think it is difficult to consider this was a recurrent lesion.

Although, the radiation dose administered was unknown, but it was obviously large enough to cause an acute radiation-induced ulcer from the patient’s complaints that the radiated postoperative wound took one year to heal [13].

Three fistulas formed at the margins of the flap around the pericardium, and the carcinoma might have developed within or near the pericardial region (Fig. 2). The cause of death in the present case was presumed to be the invasion of carcinoma into the right ventricle or superficial blood vessels of the heart.

A typical thoracic radiation-induced ulcer presents as a deep ulcer with necrotic tissue in the center of pigmented or depigmented skin.

The patient's ulcer was severe enough to expose the lungs and pericardium and was atypical. Earlier hospital biopsies, which avoided the lungs and pericardium failed to detect carcinoma.

We initially suspected a mesh-related infection and performed mesh-removal operation. At the time, the infection seemed to be localized on the mesh, and the wound did not include any structures that were located deeper than the site of the first operation.

We need to reflect on the fact that a thorough histological examination was not performed in the current case, as all ulcers can contain tumors [8,9]. However, in such cases performing a preoperative lung and pericardial biopsy under local anesthesia might not be appropriate, as it can cause bleeding, or bronchial fistula.

We should have asked thoracic or heart surgeon to perform a biopsy of the pericardium or myocardium at the time of the debridement and sent the surgical specimen for pathological evaluation.

We consulted a cardiac surgeon and asked him: If SCC had been present in the pericardium region, would it have been possible to remove it.

His opinion was that preoperative computed tomography (Fig. 1b) did not show any apparent pericardial infiltration. In addition, he stated that if the tumor had been confined to the pericardium and there had been space between it and the myocardium then the tumor could have been removed. On the other hand, if the tumor had invaded the myocardium complete resection would not have been possible, and resection avoiding the coronary arteries and veins would have been the only possible option. However, after the resection procedure it would have been necessary to cover the surface of the heart with a flap.

In conclusion, in the present case SCC developed in a chronic radiation-induced chest wall ulcer. In such cases, it is necessary to consider the best method for performing the most thorough histological examination possible, even in areas where histological examinations are difficult, as any ulcer can contain a tumor.

## Declaration of Competing Interest

There are no conflicts of interest.

## Sources of funding

There were no sources of funding support for this study except for English check fee that was paid by dermatology department in our university.

None of the authors have conflicting financial interests.

## Ethical approval

Japanese Ethical Guidelines for Medical Research for Humans.

https://www.mhlw.go.jp/file/06-Seisakujouhou-10600000-Daijinkanboukouseikagakuka/0000153339.pdf.

Treatment of this patient complies with the Japanese insurance system and does not include clinical trials, experimental interventional treatments or drug use.

This is not a medical procedure that the ethics committee or IRB should discuss.

## Consent

Written informed consent was not obtained from the patient. The head of our medical team has taken responsibility that exhaustive attempts have been made to contact the family and that the paper has been sufficiently anonymised not to cause harm to the patient or their family. A copy of a signed document stating this is available for review by the Editor-in-Chief of this journal on request.

## Author contribution

Authors’ roles:

Masamitsu Kuwahara, MD: surgeon, performed operation, wrote this manuscript.

Satoshi Yurugi, MD: assistant surgeon.

Junji Ando, MD: assistant surgeon.

Mika Takeuchi, MD: doctor in charge.

Riyo Miyata, MD: doctor in charge.

Masayuki Harada: doctor in charge.

Yasumitsu Masuda, MD: doctor in charge.

Saori Kanagawa, MD: doctor in charge.

## Registration of research studies

1. Name of the registry: this is not research paper and not registered
2. Unique identifying number or registration ID: no ID
3. Hyperlink to your specific registration (must be publicly accessible and will be checked):

## Guarantor

Masamitsu Kuwahara.

## Provenance and peer review

Not commissioned, externally peer-reviewed.
